# GNN-MA: Soft Molecular Alignment with Cross-Graph Attention for Ligand-Based Virtual Screening

**DOI:** 10.3390/molecules31060991

**Published:** 2026-03-16

**Authors:** Keling Liu, Dongmei Wei, Rui Shi, Zhiyuan Zhou

**Affiliations:** College of Computer and Software Engineering, Xihua University, Chengdu 610039, China

**Keywords:** virtual screening, scaffold hopping, molecular representation, graph attention, early enrichment

## Abstract

Ligand-based virtual screening (LBVS) seeks strong early enrichment when searching ultra-large libraries, but practical screening often relies on 1D/2D descriptions while 3D information is expensive and uncertain due to conformer generation and alignment. We propose GNN-MA, a retrieval-style pairwise scoring model for query–candidate molecular pairs that uses molecular graphs as a unified representation. Built on intra-graph message passing, GNN-MA adds cross-graph attention to learn atom-level soft alignment that focuses on key substructures relevant to activity matching, and introduces a bond-to-atom semantic aggregation module to better exploit chemical bond cues for similarity scoring. The framework uses 2D molecular graphs derived from SMILES for retrieval-style matching and does not rely on explicit 3D conformational modeling or alignment. Experiments on DUD-E and LIT-PCBA show that GNN-MA achieves competitive overall discrimination (ROC-AUC) and, relative to its ablated variants, provides consistent gains in early-enrichment metrics (EF@1–5%) on DUD-E, while on LIT-PCBA the improvements are more target-dependent. The learned atom-level soft alignment also provides a qualitative interpretability cue in case studies. Throughput benchmarks suggest that GNN-MA is most suitable as a re-ranking/refinement model after a fast prefiltering stage.

## 1. Introduction

Ligand-based virtual screening (LBVS) is a widely used prioritization strategy in early-stage drug discovery [1,2,3,4,5,6]. Its goal is to identify potential actives as early as possible from ultra-large candidate libraries [7,8,9,10,11]. Because experimental follow-up typically covers only a small top-ranked subset of compounds (often 0.1–5%), early enrichment often reflects practical screening value better than overall ranking performance [2,12,13,14,15,16]. Meanwhile, inputs in real screening workflows are frequently incomplete and heterogeneous: in most cases only 1D/2D descriptions are available, and 3D-related information is not always accessible; even when it is, conformer generation, conformer selection, and spatial alignment introduce extra cost and uncertainty [13,17,18,19,20,21,22]. Therefore, in this work we focus on a 2D graph-based formulation that avoids explicit 3D conformational modeling while still aiming at stable early enrichment under realistic screening conditions [1,2,6,7,8,9,10,11,15,16].

Existing LBVS methods can be roughly grouped into three categories [1,2,5,6,23]. The first category consists of traditional approaches built on 2D fingerprints and similarity measures, such as MACCS [24] and ECFP4 [25], which are computationally efficient and easy to scale; however, these representations inevitably compress structural information and struggle to explicitly capture fine-grained correspondences between a query and a candidate molecule, so early enrichment can be limited when the two molecules differ substantially in scaffold, or when activity is driven by only a few critical local fragments [2,6,23,24,25,26,27]. The second category includes 3D shape- or pharmacophore-matching methods (e.g., PheSA [19] and ROSHAMBO [20]), which can describe spatial similarity more directly; yet they typically rely on upstream procedures such as conformer generation and alignment, increasing computational cost and making performance sensitive to conformer quality and input settings, which in turn restricts their stable use on ultra-large libraries or in scenarios where 3D information is missing [9,12,13,17,18,19,20,28]. The third category comprises deep learning approaches to molecular representation learning [29,30,31,32,33,34,35,36,37,38,39,40], especially graph neural networks (GNNs) and models that incorporate 3D geometric information (e.g., SchNet and DimeNet++), which can learn richer structural semantics; nevertheless, many of these methods are primarily designed for single-molecule property modeling and pay insufficient attention to the query–candidate retrieval-style matching required by LBVS [3,41,42,43,44,45,46]. As a result, there is still clear room to improve how we explicitly model local correspondences and cross-molecule interactions—without relying on rigid alignment—so that the learning objective better serves early-enrichment-oriented screening and provides chemically meaningful rationales for prioritization [1,2,23,26,27,47,48,49].

To address these issues, we explicitly formulate LBVS as a query–candidate pairwise scoring-and-ranking problem and propose GNN-MA. The method uses the molecular graph as a unified representation: it first learns structural semantic embeddings via intra-molecular message passing, and then introduces a cross-graph attention mechanism to model inter-molecular interactions explicitly at the atom level. This enables atom-wise cross-molecule matching that highlights the key substructures most responsible for activity transfer and ranking decisions, providing a qualitative cue for interpreting which local fragments contribute to the matching score in case studies [3,26,44,45,46,48,49,50]. Rather than claiming novelty from the use of cross-graph attention alone, our main technical contribution lies in adapting alignment-aware graph comparison specifically to LBVS. In particular, the proposed framework combines bond-aware representation enhancement through edge fusion and bond-to-atom aggregation with a ranking-oriented training objective that is explicitly designed to improve early enrichment in target-specific virtual screening. In addition, we enhance atom representations through semantic aggregation of chemical bond information, strengthening the expressiveness of similarity estimation [41,42,43,48,50]. Importantly, GNN-MA operates on standard 2D molecular graph representations and does not depend on rigid 3D alignment procedures [2,6,13,17,18,19,20,42,43].

The main contributions of this work are as follows:

- We cast ligand-based virtual screening as a retrieval-style query–candidate pairwise scoring task, aligning model learning more closely with early-enrichment-oriented screening objectives.
- We propose an alignment-aware graph matching mechanism based on cross-graph attention to capture atom-level correspondences between molecules in a fully 2D representation space.
- We design a bond-aware and ranking-oriented learning framework by combining edge fusion, bond-to-atom aggregation, and a within-target ranking constraint for improved early retrieval.
- We provide a practical empirical study on DUD-E and LIT-PCBA with macro-aware aggregation views, per-target analysis, and efficiency evaluation for shortlist re-ranking.

## 2. Results and Discussion

### 2.1. Overview of Evaluation

We evaluated GNN-MA on two widely used ligand-based virtual screening (LBVS) benchmarks, DUD-E and LIT-PCBA. Performance was assessed using ROC-AUC [51] for overall discrimination and early enrichment EF@k% (k = 1, 2, 5, 10, 20) for top-ranked retrieval quality.

To avoid ambiguous reporting and to address target-size imbalance, we report results under four complementary aggregation views (definitions in Section 3.6): (i) global average (pooled), (ii) macro-average, (iii) weighted macro-average, and (iv) macro-target statistics. Because global average results can be disproportionately influenced by a few large targets, the main text focuses on macro-average, weighted macro-average, and macro-target evidence, whereas the corresponding global average summaries, detailed per-target tables, and supplementary statistical results are provided in the Supporting Information.

### 2.2. Results on DUD-E

#### 2.2.1. Overall Discriminative Performance (ROC-AUC)

**Figure 1:**
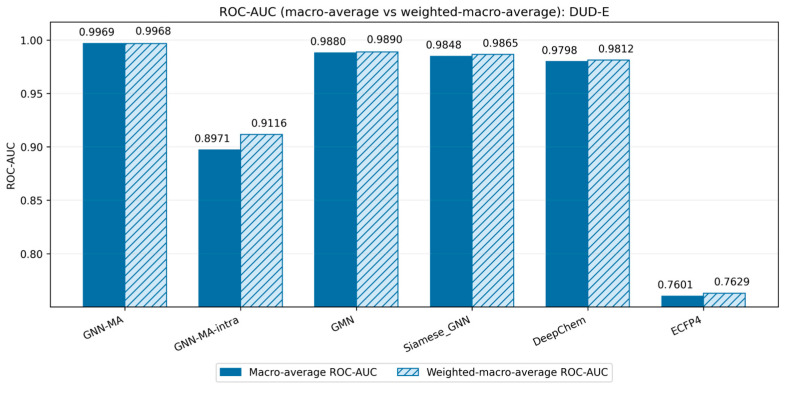
ROC-AUC comparison for DUD-E under macro-average and weighted macro-average views for each model.

Figure 1 compares ROC-AUC on DUD-E across six models under the macro-average and weighted macro-average views. GNN-MA achieves the best overall discriminative performance, while GNN-MA-intra shows a clear drop, indicating that cross-graph interaction contributes positively to discrimination. GMN [52], Siamese_GNN [53], and DeepChem provide strong learning-based baselines but remain below GNN-MA, whereas ECFP4 performs substantially worse. The close agreement between macro and weighted macro AUC indicates that the ranking of methods is stable under these macro-based summaries.

#### 2.2.2. Early Enrichment Performance (EF@k%) on DUD-E

Table 1 and Table 2 show that GNN-MA delivers stable early enrichment performance on DUD-E in both weighted macro-average and macro-average views. Its advantage is not limited to a single cutoff: the model remains strong from EF@1% through EF@20%, indicating that the gain is sustained across practical shortlist sizes rather than confined to only the very top-ranked molecules. The gap relative to GNN-MA-intra is consistent across all reported cutoffs, supporting the contribution of cross-graph interaction to retrieval quality. Compared with GMN, Siamese_GNN, and DeepChem, GNN-MA stays at the top or within the top tier under both aggregation views, whereas ECFP4 remains clearly inferior.

### 2.3. Results on LIT-PCBA

#### 2.3.1. Discriminative Performance (ROC-AUC)

**Figure 2:**
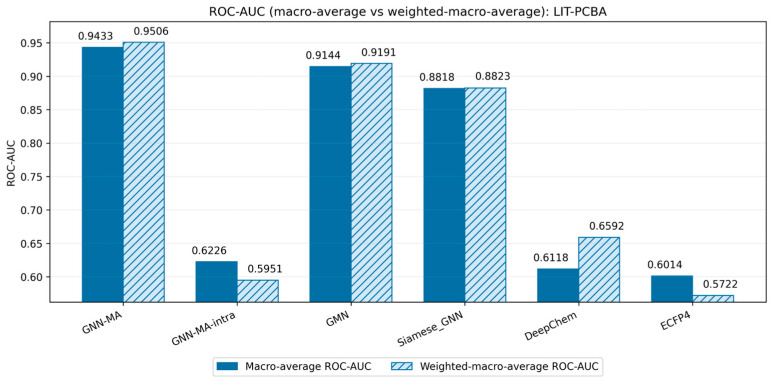
ROC-AUC comparison for LIT-PCBA under the macro-average and weighted macro-average views for each model.

Figure 2 indicates that GNN-MA maintains competitive discriminative performance on the more challenging LIT-PCBA benchmark and retains a clear advantage over GNN-MA-intra. GMN, Siamese_GNN, and DeepChem also remain competitive, whereas ECFP4 performs less favorably. Given the substantial variation in target sizes in LIT-PCBA, we focus primarily on macro-based summaries in the main text.

#### 2.3.2. Early Enrichment Performance (EF@k%) on LIT-PCBA

Table 3 and Table 4 indicate that on the more heterogeneous LIT-PCBA benchmark, the benefit of GNN-MA is most pronounced at very early cutoffs. Under both weighted macro-average and macro-average summaries, the largest margins over GNN-MA-intra appear at EF@1–5%, which is particularly relevant for top-priority retrieval in practical screening. This pattern is consistent with the stronger target-size imbalance of LIT-PCBA, where macro-based summaries are more informative than pooled results and performance is more target-dependent than on DUD-E. Relative to GMN, Siamese_GNN, and DeepChem, GNN-MA remains highly competitive across cutoffs, while ECFP4 again shows clearly weaker enrichment.

### 2.4. Per-Target Consistency Analysis (Macro)

**Figure 3:**
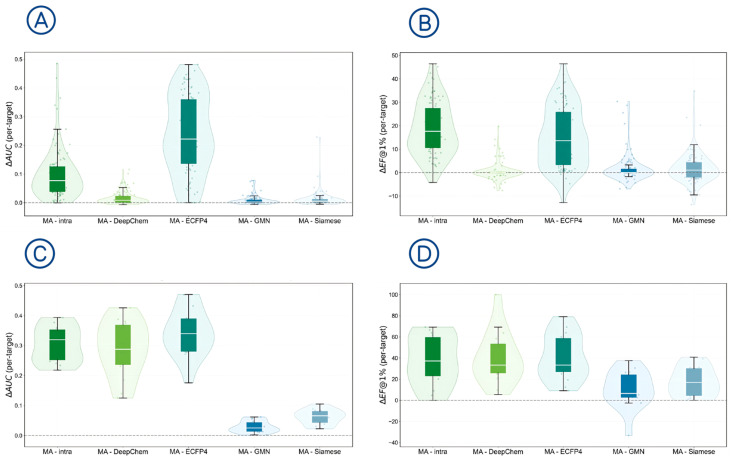
Per-target ΔAUC and ΔEF@1% distributions.

To further examine whether the observed performance gains could be consistently reflected across targets, we analyzed the per-target differences between GNN-MA and several baseline models, as illustrated in Figure 3. For each target, ROC-AUC and EF@1% were computed independently on the target-specific test set, and the differences between GNN-MA and the corresponding baselines were then calculated. The resulting ΔAUC and ΔEF@1% values therefore indicate how much GNN-MA improves or decreases performance relative to a baseline for individual targets.

Figure 3A,B show the distributions of per-target improvements for DUD-E. where green denotes comparisons against DeepChem and the intra-graph variant (GNN-MA-intra), and blue denotes comparisons against GMN and Siamese_GNN. Figure 3A presents ROC-AUC improvements, and Figure 3B presents EF@1%

The distributions are generally shifted toward positive values when comparing GNN-MA with DeepChem and the intra-graph variant (GNN-MA-intra), indicating that the performance gains are broadly observed across targets rather than driven by a small number of cases. In contrast, the differences relative to GMN and Siamese_GNN are smaller, suggesting that these graph-pair baselines remain competitive while GNN-MA maintains favorable target-level consistency.

Figure 3C,D present the corresponding results for LIT-PCBA. Because target sizes in LIT-PCBA are highly imbalanced (see Supplementary Materials Table S2), per-target analysis is particularly informative. The distributions suggest a generally positive trend in EF@1% relative to the baselines, while ROC-AUC remains broadly competitive at the target level.

### 2.5. Efficiency and Scalability

In large-scale virtual screening, inference efficiency is as critical as accuracy. To quantify deployability in large-library retrieval scenarios, we measured throughput under different CPU/GPU settings and separated preprocessing time from model inference time (Table 5).

The results show that (as shown in Table 6), in the Forward-only (pure forward inference) setting, GPU reached 280.2 pairs/s, far higher than CPU’s 9.1 pairs/s, indicating that the forward computation is highly accelerator-friendly. In the end-to-end (E2E) evaluation, enabling caching boosted GPU throughput to 231.6 pairs/s, notably higher than 161.4 pairs/s under the Online mode, suggesting that preprocessing and data pipeline overheads are key factors for E2E performance. On CPU, E2E throughput was about 8–9 pairs/s, close to its Forward-only results, implying that CPU E2E is mainly constrained by overall compute.

Overall, caching and batching can substantially improve end-to-end throughput, supporting the use of GNN-MA as a refinement model for shortlist re-ranking in large-library screening workflows.

### 2.6. Soft-Alignment Visualization

**Figure 4:**
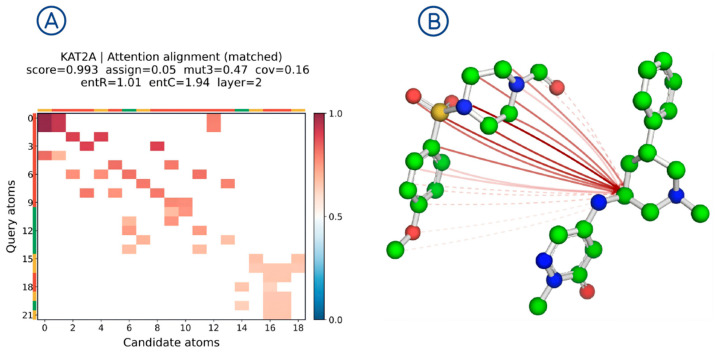
Soft-alignment visualization example: attention heatmap and atom-level matching links.

To qualitatively illustrate the matching patterns learned by the model, we present a soft-alignment case study using a query–candidate pair from the KAT2A target in DUD-E. Figure 4A visualizes attention weights between atom pairs: instead of spreading across the entire matrix, high weights concentrate in a few regions, suggesting the model tends to lock onto key fragments during matching.

Figure 4B illustrates a structural alignment sketch: the highest-weight atom correspondences from Figure 4A are mapped back onto the molecular structures and annotated with links, where darker/more solid lines indicate higher weights. Different atom colors follow the standard molecular visualization convention and denote different element types. The links cluster around a few crucial local regions rather than scattering randomly. This pattern indicates that cross-graph attention highlights a small number of locally focused correspondences, which can be qualitatively inspected to understand which fragments contribute most to the matching score in this example.

### 2.7. Summary

In summary, this chapter provides a systematic evaluation of GNN-MA from five aspects: overall performance, early enrichment, cross-target robustness, inference efficiency, and qualitative alignment visualization. Taken together, the results indicate that GNN-MA combines solid discriminative performance with stronger very-early retrieval behavior, while the per-target and efficiency analyses further clarify where these advantages are most evident and how the model may be used in practical re-ranking settings. Meanwhile, per-target analysis and the visualization case study provide qualitative support for the model’s learned matching behavior. Throughput benchmarking also suggests practical deployability, and indicates that caching and batching can markedly improve end-to-end screening efficiency.

## 3. Materials and Methods

### 3.1. Study Overview and Problem Definition

We formulated ligand-based virtual screening (LBVS) as a query–candidate pairwise scoring task. Given a query molecule $G_{q}$ and a candidate molecule $G_{c}$ under the same target, we learned a differentiable scoring function $S(G_{q},\;G_{c})$ so that active compounds in the candidate library receive higher scores and are ranked earlier.

Training data were constructed in pairs: ligand–active pairs were treated as positives, and ligand–decoy/inactive pairs as negatives. The model output a continuous matching score to rank candidates, and we reported ROC-AUC and early-enrichment metrics (EF@k%) during testing.

### 3.2. Datasets and Preprocessing

#### 3.2.1. Datasets and Molecular Representation

We evaluated our method on two standard LBVS benchmarks: DUD-E [14] and LIT-PCBA [16]. Both datasets are organized by target, and each target contains active molecules and inactive molecules. DUD-E originally included 102 targets; after removing 2 targets that failed parsing, we retained 100 targets. LIT-PCBA contains 15 targets, and all were used in this study.

Molecules were represented as 2D heavy-atom graphs derived from SMILES. We did not impose a hard limit on the number of atoms; variable-sized molecules were handled via in-batch padding and masking.

#### 3.2.2. Data Split and Evaluation Protocol

To ensure fairness, reproducibility, and leakage-free evaluation, we adopted a unified molecule-level splitting and evaluation protocol.

(1) Molecule-level split. For each target *t*, molecules were split into train/validation/test subsets with a ratio of 8:1:1 at the molecule level. The split satisfies disjointness:

(1) $$M_{t}=M_{t}^{train}\cup M_{t}^{val}\cup M_{t}^{test}\;$$

(2) $$M_{t}^{train}\cap M_{t}^{val}=M_{t}^{train}\cap M_{t}^{test}=M_{t}^{val}\cap M_{t}^{test}=\emptyset$$

A fixed random seed was used and the same split was reused across all experiments. All models in this work were trained and evaluated under an identical partition.

(2) Pair construction. Within each target, ligand–active pairs form positive samples and ligand–decoy/inactive pairs form negative samples. Pair construction strictly respected subset boundaries, preventing pair-level leakage. During evaluation, all molecules used for scoring were taken exclusively from the test split of each target. Pairwise scores were computed only between molecules within the same target-specific test subset, and the resulting scores were ranked to compute ROC-AUC and EF@k%.
(3) Evaluation views. During testing, ranking was performed independently within each target. We report: (i) pooled metrics that aggregate per-target statistics; and (ii) per-target metrics and robustness analysis. Formal metric definitions are provided in Section 3.6.

### 3.3. Molecular Graph Representation and Features

We represented a molecule as a graph G=(V,E), where $V\in R^{N\times d_{n}}$ is the atom feature matrix (with $d_{n}$ as the node feature dimension), and $E\in R^{N\times N\times d_{e}}$ is the bond feature tensor (with $d_{e}$ as the edge feature dimension). To reflect the incomplete and heterogeneous inputs in real-world LBVS, we adopted a dimension-agnostic feature organization strategy: we used 2D topology and atom/bond attributes as the input features throughout all experiments. The specific atom and bond features we used are listed in Table 7 and Table 8.

### 3.4. GNN-MA Model Architecture

**Figure 5:**
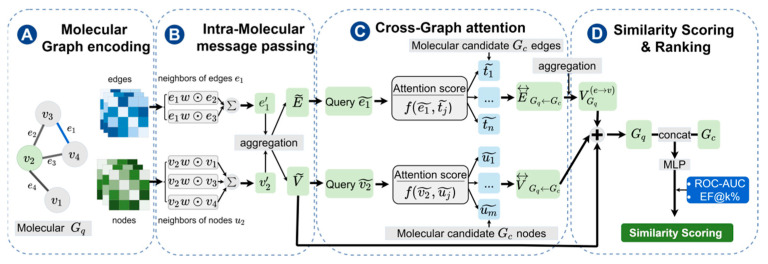
Architecture of GNN-MA.

As illustrated in Figure 5, GNN-MA comprises four components: (i) Intra-Graph Message Passing, (ii) Cross-Graph Attention for Soft Alignment, (iii) Edge Fusion and Bond-to-Atom Aggregation, and (iv) graph-level pooling followed by an MLP to output the final pairwise matching score.

#### 3.4.1. Intra-Molecular Message Passing

To learn intra-molecular structural semantics, GNN-MA performs l-th layer message passing on the query graph $G_{q}$ and the candidate graph $G_{c}$ separately. Given the (l-1)th layer node embedding $V_{i}^{(l-1)}$ and the edge (bond) representation $e_{ij}^{(l-1)}$ between nodes i and j, node i aggregates messages from its neighborhood N(i) as follows:

(3) $$m_{i}^{\left(l\right)}\;=\;\sum_{j\in N\left(i\right)}\psi^{\left(l\right)}(v_{i}^{(l-1)},v_{j}^{(l-1)},e_{ij}^{(l-1)})$$

The node embeddings are then updated via the following function:

(4) $$v_{i}^{\left(l\right)}\;=\;\phi^{\left(l\right)}(v_{i}^{(l-1)},m_{i}^{\left(l\right)})$$

Here, $\psi^{\left(l\right)}$ and $\psi^{\left(l\right)}$ are learnable mappings (e.g., multilayer perceptrons) that integrate neighborhood atom and bond information into the node representation. To improve training stability and representation capacity, we further apply residual connections and normalization between layers.

#### 3.4.2. Cross-Graph Attention for Soft Alignment

Independent encoding of each molecule is typically insufficient to extract the pairwise matching cues required for retrieval. Therefore, GNN-MA builds upon the intra-molecular representations with a cross-graph attention module that explicitly captures atom- and bond-level interactions between the query and candidate, producing an explicit soft-alignment matrix.

Let $V_{q}\;=\;[v_{q,1},\ldots ,v_{q,n}]\in R^{n\times d}$ and $V_{c}\;=\;[v_{c,1},\ldots ,v_{c,m}]\in R^{m\times d}$, denoting the atom representations of the two compounds after intra-molecular message passing, where n and m are the numbers of atoms and d is the embedding dimension. We use scaled dot-product attention to compute the cross-graph relevance between the i-th atom in the query molecule $G_{q}$ and the j atom in the candidate molecule $G_{c}$:

(5) $$\alpha_{ij\;}=\;\frac{\left(W_{q}v_{q,i}{)}^{T}(W_{c}v_{c,j}\right)}{\sqrt{d}}$$

Here, $W_{q}$ and $W_{c}$ are learnable parameters, and $\sqrt{d}$ is the scaling factor used in the attention mechanism.

For each query atom i, we normalize across all candidate atoms j by applying a softmax along the candidate atom dimension, yielding the soft alignment weights from query to candidate:

(6) $$A_{q\to c}(i,j)\;=\;{\mathrm{softmax}}_{j}(\alpha_{ij})$$

Based on these weights, query atom i aggregates information from the candidate molecule to obtain a cross-graph contextual representation:

(7) $${\tilde{V}}_{q,i}=\sum_{j=1}^{m}A_{q\to c}(i,j)\;v_{c,j}$$

To enhance the symmetry of alignment and the complementarity of information, we similarly compute the reverse attention $A_{c\to q}$ and obtained ${\tilde{V}}_{c,j}$.

Analogously, we also construct cross-graph attention at the bond level: the correlation computation, normalization, and cross-graph aggregation follow the same procedure as the atom-level attention, except that the inputs are changed from atom representations to bond representations.

#### 3.4.3. Edge Fusion and Bond-to-Atom Aggregation

The atom-level interaction representations produced by cross-graph attention provide evidence of soft correspondences between the query and candidate. Building on this, GNN-MA introduces a two-stage structural enhancement prior to node updating, namely edge-level fusion → edge-to-node aggregation: we first fuse information at the bond (edge) level to obtain enhanced edge representations, and then aggregate these enhanced edge features to adjacent atoms. In this way, bond semantics are injected into atom representations and subsequently contribute to the final scoring.

(1) Edge-level Fusion:

For each chemical bond, we construct a fused edge representation by combining the cross-graph-updated representations of its two incident atoms with the original bond feature:

(8) $${\hat{e}}_{ij}\;=\;\mathrm{EdgeFuse}(v_{i},v_{j},e_{ij})$$

Here, EdgeFuse(·) is a learnable mapping that integrates node semantics and bond information to produce an enhanced bond representation.

(2) Edge-to-node aggregation

After obtaining the fused edge representations, we aggregate information from incident edges for each atom to form an edge-aggregation vector:

(9) $$g_{i}\;=\;\sum_{j\in N\left(i\right)}e_{ij}$$

Then, we inject the aggregated bond information into the atom representation through a learnable transformation and an update operation:

(10) $$V^{\left(e\to v\right)}\;=\;\sum_{i\in V}\eta (g_{i})$$

Here, η(·) is a learnable mapping that transfers bond-level information to atom-level representations, facilitating subsequent fusion with atom features.

This design is particularly relevant to LBVS, where subtle local bond environments can affect functional similarity even when global graph topology appears similar.

#### 3.4.4. Similarity Scoring and Ranking

For graph-level pooling, we fuse the molecular representations obtained from intra-graph convolution, cross-graph attention, and edge-to-node aggregation via a residual combination, and then apply a readout function to obtain graph-level embeddings for scoring:

(11) $$Z_{q}\;=\;pool({\tilde{V}}_{q}\;+{\overset{⃡}{\;V}}_{q}\;+{\;V}_{q}^{(e\to v)}),\;{\;Z}_{c}\;=\;pool({\tilde{V}}_{c}\;+{\overset{⃡}{\;V}}_{c}\;+{\;V}_{c}^{(e\to v)})$$

For pairwise scoring, we combine the two graph-level embeddings and feed them into a multilayer perceptron to obtain the final matching score:

(12) $$S(G_{q},G_{c})\;=\;MLP(Z_{q}\;||\;{\;Z}_{c})$$

Here, || denotes feature concatenation.

First, the model outputs a matching score for each query–candidate molecular pair. During training, we used the binary cross-entropy loss as the primary supervision signal for active/decoy classification. To further emphasize early enrichment, we added a within-target batch-wise ranking constraint. Specifically, each mini-batch was constructed from molecules belonging to the same target. Within the current mini-batch, negative pairs are ranked according to their predicted similarity scores, and the top K highest-scoring negatives are selected as the hardest negatives (K = 10). The ranking term then encourages the scores of positive pairs to be higher than those of these selected hard negatives. In this work, the final objective is defined as:

(13) $$L\;={\;L}_{bce}\;+\;\lambda L_{rank}$$

where L_bce_ denotes the binary cross-entropy loss and L_rank_ denotes the batch-wise pairwise ranking loss. To stabilize training, the ranking term is activated only after a short warm-up period: λ = 0 for the first two epochs and λ = 0.05 for the remaining epochs.

Compared with a pure pairwise classification objective, this ranking-oriented design better reflects the practical goal of LBVS, namely prioritizing active candidates at the top of the ranked list.

### 3.5. Baselines and Ablation Settings

To assess how a general 2D molecular graph classifier performs at this task, we chose DeepChem’s GraphConvModel [46,54], GMN [52], Siamese_GNN [53], and ECFP4 [25] as representative comparison methods. GraphConvModel serves as a general molecular graph classification baseline, learning structural representations directly from 2D molecular graphs and producing a binary classification score. GMN and Siamese_GNN provide pairwise graph-matching and similarity-learning baselines, respectively, while ECFP4 serves as a classical fingerprint-based method. On both DUD-E and LIT-PCBA, all baselines were evaluated using the same data split and evaluation pipeline as our main model, and we report both pooled and per-target AUC and EF@k%. We also built an ablated variant, GNN-MA-intra. It matches GNN-MA exactly in data splits, loss functions, and hyperparameter settings, but removes the cross-graph attention module, ensuring a fair and reproducible comparison.

### 3.6. Evaluation Metrics and Statistical Reporting

#### 3.6.1. ROC-AUC

Overall ranking quality was evaluated using the area under the receiver operating characteristic curve [51] (ROC-AUC). ROC-AUC measures the probability that a randomly selected active molecule receives a higher predicted score than a randomly selected inactive molecule. It is threshold-independent and reflects global discriminative ability.

#### 3.6.2. Early Enrichment: EF@k%

To assess early retrieval performance under realistic screening constraints, we report the enrichment factor EF@k% [55] for k = 1, 2, 5, 10, and 20. For target t, let $N_{t}$ denote the number of test molecules and $A_{t}$ the number of test actives. After ranking molecules by predicted score in descending order, the top-k% cutoff is defined as:

(14) $$n_{t}^{k}=\frac{k}{100}N_{t}$$

Let $a_{t}^{k}$ denote the number of actives within the top $n_{t}^{k}$ molecules. The target-wise enrichment factor is defined as:

(15) $${EF}_{t}(k)=\frac{a_{t}^{k}/n_{t}^{k}}{A_{t}/N_{t}}$$

We report k∈{1,2,5,10,20} with particular emphasis on EF@1%, which reflects very early enrichment.

#### 3.6.3. Aggregation Views Across Targets

Because targets can differ substantially in candidate-set size and active ratio, we report four complementary aggregation views:

(1) Global average (pooled)

Predictions from all targets are pooled together before computing the metric. This view provides a single overall summary and aligns with pooled reporting conventions in many prior works. However, it can be dominated by a few large targets.

(2) Macro-average

Metrics are computed independently for each target and then averaged with equal weights across targets. This view reflects cross-target robustness by treating each target equally.

(3) Weighted macro-average

Per-target metrics are averaged with weights proportional to the number of evaluated molecules (or candidates) per target. This view lies between macro-average and global average and helps assess whether aggregate results are driven by a target-size imbalance.

(4) Macro-target statistics

Metrics are analyzed at the target level to assess consistency across targets. We summarize per-target performance using distributions, win/loss/tie counts, and paired statistical tests on per-target differences.

#### 3.6.4. Per-Target Improvement and Significance Testing

For a given metric M (ROC-AUC or EF@1%), we defined the per-target improvement over a baseline as

(16) $$\Delta_{t}=M_{t}(GNN\_MA)-M_{t}(baseline)$$

We estimated uncertainty of the mean improvement using a nonparametric bootstrap over targets (B = 20,000 resamples) and report percentile-based 95% confidence intervals.

To test whether improvements are systematically positive across targets, we applied a two-sided Wilcoxon signed-rank test to the set of per-target differences {$\Delta_{t}$}. We additionally report win/loss/tie counts, where a win indicates $\Delta_{t}$ > 0, a loss indicates $\Delta_{t}$ < 0, and a tie indicates $\Delta_{t}$ = 0.

In the main text, EF tables are reported under macro-average and weighted macro-average views, whereas global average metrics are included as supplementary results.

### 3.7. Implementation Details and Throughput Benchmark

We implemented the model in PyTorch 2.6.0+cu126 and used Adam optimizer [56] for optimization. Key hyperparameters were: batch size = 32; epochs = 20; learning rate = 1 × 10^3^; weight decay = 1 × 10^−4^; dropout = 0.2; warm-up = 2; model size: hidden dim = 64; message passing layers = 3; gradient clipping max-norm = 5.0.

Hardware and software environment: OS = Windows 10 (10.0.26100); Python 3.11.9; PyTorch 2.6.0+cu126; CUDA 12.6; GPU = NVIDIA GeForce RTX 4060 Ti; CPU = Intel Core i5-14600K; RAM = 32 GB.

For the ranking loss, we set K = 10 hardest negatives per batch and λ = 0.05 after a two-epoch warm-up period. These values were kept fixed across all experiments.

## 4. Conclusions

We propose GNN-MA, a soft molecular alignment approach for ligand-based virtual screening (LBVS). Using molecular graphs as a unified representation, GNN-MA learns atom-level correspondences via cross-graph attention and, together with intra-graph message passing and bond-to-atom aggregation, explicitly injects cross-molecule matching evidence into similarity scoring and provides a qualitative interpretability cue in case studies.

Experiments on DUD-E and LIT-PCBA support the effectiveness of the proposed alignment-aware design: cross-graph interaction improves retrieval quality relative to the intra-graph variant, particularly in the early-ranking regime, while the accompanying per-target and throughput analyses suggest that the model is best suited for shortlist refinement rather than brute-force large-library screening. Throughput benchmarking further suggests strong deployment potential, showing that caching and batching can substantially improve end-to-end screening efficiency.

Future work will focus on more robust early-ranking objectives, improved negative sampling strategies, and validation on additional external benchmarks.

## Figures and Tables

**Table 1:** DUD-E early enrichment EF@k% under the weighted macro-average view.

Model	EF@1%	EF@2%	EF@5%	EF@10%	EF@20%
GNN-MA	35.52	34.54	19.42	9.89	4.98
GNN-MA-intra	17.51	15.52	10.94	7.24	4.28
DeepChem	35.30	32.45	17.79	9.39	4.85
GMN	34.24	32.16	18.16	9.63	4.94
Siamese_GNN	33.39	31.39	18.04	9.60	4.93
ECFP4	19.35	14.05	7.77	4.73	2.99

**Table 2:** DUD-E early enrichment EF@k% under the macro-average view.

Model	EF@1%	EF@2%	EF@5%	EF@10%	EF@20%
GNN-MA	34.38	33.20	19.26	9.86	4.98
GNN-MA-intra	15.74	14.61	10.20	6.89	4.14
DeepChem	33.81	31.55	17.45	9.34	4.83
GMN	32.58	30.91	17.91	9.59	4.93
Siamese_GNN	32.03	30.53	17.75	9.57	4.93
ECFP4	19.05	14.00	8.08	4.79	3.00

**Table 3:** LIT-PCBA early enrichment EF@k% under the weighted macro-average view.

Model	EF@1%	EF@2%	EF@5%	EF@10%	EF@20%
GNN-MA	55.94	32.10	15.47	8.60	4.65
GNN-MA-intra	9.89	6.97	3.36	2.21	1.87
DeepChem	6.91	6.86	3.95	2.65	1.96
GMN	36.66	23.95	12.06	7.49	4.40
Siamese_GNN	33.50	20.68	11.02	6.58	3.88
ECFP4	5.74	5.39	2.33	1.78	1.42

**Table 4:** LIT-PCBA early enrichment EF@k% under the macro-average view.

Model	EF@1%	EF@2%	EF@5%	EF@10%	EF@20%
GNN-MA	44.55	29.64	14.95	8.21	4.57
GNN-MA-intra	6.98	4.95	2.9	2.3	1.84
DeepChem	4.61	4.46	3.26	2.14	1.64
GMN	33.73	21.82	12.44	7.47	4.31
Siamese_GNN	26.8	18.68	10.32	6.47	3.86
ECFP4	3.62	2.73	2.29	1.84	1.51

**Table 5:** Throughput benchmark protocol and reporting scheme.

Dimension	Level (Paper Wording)	Notation/Unit	Reporting Convention (Journal-Ready Wording)
Pipeline scope	Forward-only	Forward-only	Measures model forward pass only (including necessary tensor transfer/synchronization), excluding data loading and graph construction; reflects the upper bound of model compute efficiency.
	End-to-end	E2E	Includes molecule → graph construction/feature encoding + data loading + forward; reflects deployment-relevant wall-clock cost.
Compound preparation (E2E)	Cache	Cache	Graph/features are precomputed and cached (e.g., files in the LMDB directory). E2E primarily covers loading + forward.
	Online	Online	Graph construction/encoding is performed on the fly. E2E covers construction/encoding + loading + forward.
Hardware	GPU	GPU	Reports GPU model, memory, FP16/AMP setting, batch size, num_workers, etc.
	CPU	CPU	Reports CPU model, thread count, MKL/OMP setting, batch size, etc.
Scalability outputs	100 K/1 M time	T(10^5^), T(10^6^)	Fixed query: T(N) = N/throughput. Variable queries: T(Q,N) = Q × N/(pairs/s).
	Per-pair latency	ms/pair	t-pair = 1/(pairs/s) (converted to milliseconds).

**Table 6:** Throughput and scalability results (CPU/GPU, Forward-only vs. E2E, Cache/Online).

Pipeline Scope	Compound Prep	Hardware	Throughput (pairs/s)	Per-Pair Latency (ms/pair)	100 K Time T (10^5^)	1 M Time T (10^6^)
Forward-only	—	GPU	280.2	3.5690	356.9	3568.9
Forward-only	—	CPU	9.1	110.0933	10,989.0	109,890.1
E2E	Cache	GPU	231.6	4.3183	431.8	4317.8
E2E	Cache	CPU	8.3	120.5607	12,048.2	120,481.9
E2E	Online	GPU	161.4	6.1955	619.5	6195.5
E2E	Online	CPU	8.17	122.4379	12,243.8	122,437.9

**Table 7:** Atom features included.

No.	Feature Category	Meaning/Examples	Notes (Task Relevance)
1	Atom type	C, O, N, S, etc.	Basic chemical composition that influences molecular properties and interaction patterns.
2	Topological position	On the main scaffold/on a side chain	Distinguishes the structural core from substituents, affecting conformational flexibility and local interactions.
3	Scaffold type	Aliphatic scaffold, aromatic scaffold, etc.	Reflects global structural framework differences, often associated with hydrophobicity, rigidity, and overall molecular shape.
4	Aromaticity	Whether the atom belongs to an aromatic ring	Aromaticity affects electron distribution and pi–pi interactions, which are frequently linked to bioactivity.
5	Ring membership	Whether the atom is part of any ring system	Ring membership impacts rigidity, geometry, and accessibility, thereby influencing structural matching in screening.
6	Pharmacophoric features	H-bond donor, H-bond acceptor, aromatic ring, hydrophobic site, etc.	Captures key structural motifs closely related to ligand–target interactions and biological activity.

**Table 8:** Bond features included.

No.	Feature Category	Meaning/Examples	Notes (Task Relevance)
1	Bond type	Single, double, triple, aromatic	Determines connectivity strength and geometric/electronic structure; fundamental to molecular topology and reactivity.
2	Aromatic bond	Whether the bond is aromatic	Indicates conjugated/pi systems, affecting electron delocalization and molecular recognition (e.g., pi–pi interactions).
3	Conjugation	Whether the bond is conjugated	Related to electron delocalization, influencing polarity, stability, and interaction patterns.
4	In-ring bond	Whether the bond is within a ring	Ring bonds constrain molecular geometry and rigidity, affecting structural matching during screening.

## Data Availability

The benchmark datasets used in this study, DUD-E and LIT-PCBA, are publicly available. All data used to generate the figures and tables in this manuscript are available from the corresponding author upon reasonable request (if applicable). The training and evaluation code for GNN-MA is available in a public repository: https://github.com/BobbyLiukeling/GNN-MA (accessed on 13 March 2026).

## Supplementary Materials

The following supporting information can be downloaded at: https://www.mdpi.com/article/10.3390/molecules31060991/s1, Figure S1: DUD-E cumulative contribution curve; Figure S2: LIT-PCBA cumulative contribution curve; Figure S3: Global ROC curves for all compared models on DUD-E; Figure S4: Global ROC curves for all compared models on LIT-PCBA; Table S1: DUD-E per-target statistics; Table S2: LIT-PCBA per-target statistics; Table S3: Win/loss/tie statistics; Table S4: Global ROC-AUC results; Table S5: Global EF results; Table S6: Statistical significance of per-target improvements; Table S7: Per-target ROC-AUC and EF@1% values for all models on DUD-E and LIT-PCBA.
