# Supplementary material for: GNN-MA: Soft Molecular Alignment with Cross-Graph Attention for Ligand-Based Virtual Screening
Source: Molecules. 2026 Mar 16;31(6):991. doi: 10.3390/molecules31060991 (PMC13029554; doi:10.3390/molecules31060991)
Supplement: Supplementary file 1 [file molecules-31-00991-s001.zip › molecules-4179466-supplementary.pdf]

Supplementary Information

S1 Dataset Split and Statistics

S1.1 Split protocol

Each dataset was split at the molecule level into training, validation, and test subsets with a ratio of 8:1:1. The same predefined split files were used for all compared models to ensure fair and reproducible evaluation.

S1.2 Overlap verification

We further verified split disjointness for every target and confirmed that no molecule appeared in more than one subset (train/validation/test). This check helps exclude molecule-level leakage across data partitions.

Table S1. DUD-E per-target statistics

Target	Total	Actives	Decoys/Inactives	Train	Val	Test	%Total	%Actives	%Test
abl1	11180	295	10885	8944	1117	1119	0.79%	0.75%	0.79%
ace	17929	808	17121	14342	1792	1795	1.26%	2.05%	1.26%
aces	27037	664	26373	21629	2703	2705	1.90%	1.69%	1.90%
ada	5734	262	5472	4586	573	575	0.40%	0.66%	0.40%
ada17	37606	959	36647	30084	3759	3763	2.65%	2.43%	2.64%
adrb1	16416	458	15958	13132	1640	1644	1.16%	1.16%	1.16%
adrb2	15702	447	15255	12561	1569	1572	1.10%	1.13%	1.10%
akt1	16998	423	16575	13598	1699	1701	1.20%	1.07%	1.20%
akt2	7142	190	6952	5713	714	715	0.50%	0.48%	0.50%
aldr	9356	220	9136	7484	935	937	0.66%	0.56%	0.66%
ampc	2963	62	2901	2369	296	298	0.21%	0.16%	0.21%
andr	15026	523	14503	12020	1502	1504	1.06%	1.33%	1.06%
aofb	7099	168	6931	5678	709	712	0.50%	0.43%	0.50%
bace1	18706	485	18221	14964	1870	1872	1.32%	1.23%	1.32%
braf	10349	251	10098	8278	1034	1037	0.73%	0.64%	0.73%
cah2	32542	835	31707	26033	3253	3256	2.29%	2.12%	2.29%
casp3	11172	350	10822	8937	1117	1118	0.79%	0.89%	0.79%
cdk2	29125	798	28327	23299	2911	2915	2.05%	2.03%	2.05%
comt	4012	86	3926	3208	400	404	0.28%	0.22%	0.28%
cp2c9	7757	183	7574	6205	775	777	0.55%	0.46%	0.55%
cp3a4	12303	363	11940	9842	1230	1231	0.87%	0.92%	0.87%
csf1r	12719	286	12433	10174	1271	1274	0.89%	0.73%	0.90%
cxcr4	3535	122	3413	2827	353	355	0.25%	0.31%	0.25%
def	5899	161	5738	4718	589	592	0.42%	0.41%	0.42%
dhi1	20141	519	19622	16112	2013	2016	1.42%	1.32%	1.42%
dpp4	42451	1079	41372	33960	4244	4247	2.99%	2.74%	2.98%
drd3	35063	877	34186	28049	3505	3509	2.47%	2.23%	2.47%
dyr	17950	566	17384	14359	1794	1797	1.26%	1.44%	1.26%

Target	Total	Actives	Decoys/Inactives	Train	Val	Test	%Total	%Actives	%Test
egfr	36274	832	35442	29018	3627	3629	2.55%	2.11%	2.55%
esr1	21445	627	20818	17155	2143	2147	1.51%	1.59%	1.51%
esr2	20908	595	20313	16726	2090	2092	1.47%	1.51%	1.47%
fa10	21208	792	20416	16965	2120	2123	1.49%	2.01%	1.49%
fa7	6487	185	6302	5189	648	650	0.46%	0.47%	0.46%
fabp4	2912	57	2855	2329	290	293	0.20%	0.14%	0.21%
fak1	5515	114	5401	4411	551	553	0.39%	0.29%	0.39%
fkb1a	6105	273	5832	4883	610	612	0.43%	0.69%	0.43%
fnta	53731	1692	52039	42984	5372	5375	3.78%	4.29%	3.78%
fpps	9206	213	8993	7364	920	922	0.65%	0.54%	0.65%
gcr	15747	563	15184	12597	1574	1576	1.11%	1.43%	1.11%
glcm	4150	313	3837	3319	414	417	0.29%	0.79%	0.29%
gria2	12330	297	12033	9863	1232	1235	0.87%	0.75%	0.87%
grik1	6768	152	6616	5413	676	679	0.48%	0.39%	0.48%
hdac2	10604	238	10366	8482	1059	1063	0.75%	0.60%	0.75%
hdac8	10748	234	10514	8598	1074	1076	0.76%	0.59%	0.76%
hivint	6960	211	6749	5567	695	698	0.49%	0.54%	0.49%
hivpr	37630	1395	36235	30104	3762	3764	2.65%	3.54%	2.65%
hivrt	19773	639	19134	15818	1976	1979	1.39%	1.62%	1.39%
hmdh	9183	299	8884	7346	917	920	0.65%	0.76%	0.65%
hs90a	5067	125	4942	4053	506	508	0.36%	0.32%	0.36%
hxk4	4930	127	4803	3943	492	495	0.35%	0.32%	0.35%
igf1r	9630	226	9404	7703	962	965	0.68%	0.57%	0.68%
inha	2389	71	2318	1910	238	241	0.17%	0.18%	0.17%
ital	8923	233	8690	7138	892	893	0.63%	0.59%	0.63%
jak2	6743	153	6590	5394	674	675	0.47%	0.39%	0.47%
kif11	7108	197	6911	5685	710	713	0.50%	0.50%	0.50%
kit	10861	252	10609	8688	1085	1088	0.76%	0.64%	0.76%
kith	2998	132	2866	2397	299	302	0.21%	0.33%	0.21%
kpcb	9091	248	8843	7272	908	911	0.64%	0.63%	0.64%
lck	28539	683	27856	22830	2853	2856	2.01%	1.73%	2.01%
lkha4	9721	244	9477	7776	971	974	0.68%	0.62%	0.68%
mapk2	6450	206	6244	5159	644	647	0.45%	0.52%	0.45%
mcr	5433	193	5240	4346	543	544	0.38%	0.49%	0.38%
met	11676	244	11432	9340	1167	1169	0.82%	0.62%	0.82%
mk01	4767	139	4628	3813	475	479	0.34%	0.35%	0.34%
mk10	6900	186	6714	5519	689	692	0.49%	0.47%	0.49%
mk14	37347	915	36432	29877	3734	3736	2.63%	2.32%	2.63%
mmp13	39046	1038	38008	31236	3903	3907	2.75%	2.63%	2.75%
mp2k1	8483	242	8241	6785	848	850	0.60%	0.61%	0.60%
nos1	8307	234	8073	6645	830	832	0.58%	0.59%	0.58%
nram	6449	222	6227	5158	644	647	0.45%	0.56%	0.45%
pa2ga	5341	127	5214	4272	533	536	0.38%	0.32%	0.38%
parp1	31169	742	30427	24934	3116	3119	2.19%	1.88%	2.19%
pde5a	28530	706	27824	22823	2852	2855	2.01%	1.79%	2.01%
pgh1	11193	251	10942	8953	1119	1121	0.79%	0.64%	0.79%

Target	Total	Actives	Decoys/Inactives	Train	Val	Test	%Total	%Actives	%Test
pgh2	23936	531	23405	19148	2393	2395	1.68%	1.35%	1.68%
plk1	7034	155	6879	5627	702	705	0.49%	0.39%	0.50%
pnph	7249	233	7016	5798	724	727	0.51%	0.59%	0.51%
ppara	20375	544	19831	16299	2037	2039	1.43%	1.38%	1.43%
ppard	13520	288	13232	10815	1351	1354	0.95%	0.73%	0.95%
pparg	26589	723	25866	21270	2658	2661	1.87%	1.83%	1.87%
prgr	16258	444	15814	13006	1625	1627	1.14%	1.13%	1.14%
ptn1	7624	225	7399	6099	761	764	0.54%	0.57%	0.54%
pur2	2926	201	2725	2340	292	294	0.21%	0.51%	0.21%
pygm	4152	114	4038	3321	414	417	0.29%	0.29%	0.29%
pyrd	6782	134	6648	5425	677	680	0.48%	0.34%	0.48%
reni	7371	387	6984	5896	736	739	0.52%	0.98%	0.52%
rock1	6580	203	6377	5263	657	660	0.46%	0.52%	0.46%
rxra	7869	162	7707	6294	786	789	0.55%	0.41%	0.55%
sahh	3673	190	3483	2938	367	368	0.26%	0.48%	0.26%
src	35788	831	34957	28629	3578	3581	2.52%	2.11%	2.52%
tgfr1	8957	281	8676	7164	895	898	0.63%	0.71%	0.63%
thb	7807	168	7639	6245	779	783	0.55%	0.43%	0.55%
thrb	28182	861	27321	22544	2818	2820	1.98%	2.19%	1.98%
try1	26977	758	26219	21581	2696	2700	1.90%	1.92%	1.90%
tryb1	7884	171	7713	6306	788	790	0.55%	0.43%	0.56%
tysy	7192	311	6881	5752	719	721	0.51%	0.79%	0.51%
urok	10239	306	9933	8190	1023	1026	0.72%	0.78%	0.72%
vgfr2	25899	620	25279	20719	2589	2591	1.82%	1.57%	1.82%
wee1	6371	137	6234	5096	636	639	0.45%	0.35%	0.45%
xiap	5342	129	5213	4273	533	536	0.38%	0.33%	0.38%

Table S2. LIT-PCBA per-target statistics

Target	Total	Actives	Decoys/Inactives	Train	Val	Test	%Total	%Actives	%Test
ADRB2	312500	17	312483	249999	31249	31252	11.13%	0.17%	11.13%
ALDH1	145133	7168	137965	116106	14512	14515	5.17%	71.44%	5.17%
ESR1_ago	5596	13	5583	4476	559	561	0.20%	0.13%	0.20%
ESR1_ant	5050	102	4948	4039	504	507	0.18%	1.02%	0.18%
FEN1	355771	369	355402	284616	35576	35579	12.67%	3.68%	12.67%
GBA	296218	166	296052	236973	29621	29624	10.55%	1.65%	10.55%
IDH1	362088	39	362049	289670	36207	36211	12.89%	0.39%	12.89%
KAT2A	348742	194	348548	278993	34873	34876	12.42%	1.93%	12.42%
MAPK1	62937	308	62629	50349	6292	6296	2.24%	3.07%	2.24%
MTORC1	33069	97	32972	26454	3306	3309	1.18%	0.97%	1.18%
OPRK1	269840	24	269816	215871	26983	26986	9.61%	0.24%	9.61%
PKM2	246069	546	245523	196854	24606	24609	8.76%	5.44%	8.76%
PPARG	5238	27	5211	4189	523	526	0.19%	0.27%	0.19%
TP53	4247	79	4168	3397	423	427	0.15%	0.79%	0.15%
VDR	356272	884	355388	285017	35626	35629	12.68%	8.81%	12.68%

Per-target molecule counts and split sizes are summarized in Tables S1–S2 to contextualize target-size effects discussed later.

Target-size cumulative analysis

Figures below show the cumulative proportion of evaluation samples (test set) contributed by targets sorted in descending order of size. A steep rise indicates that a small number of large targets dominate the pooled evaluation set.

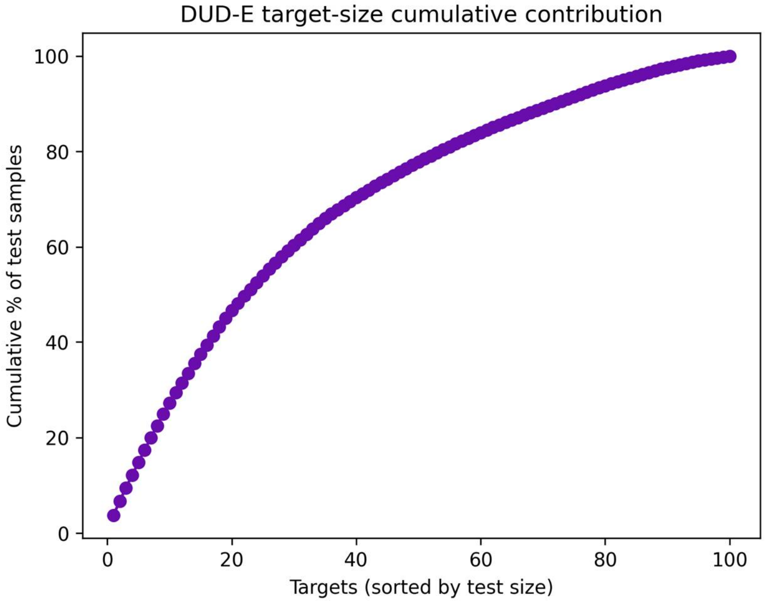

Figure S1. DUD-E cumulative contribution curve

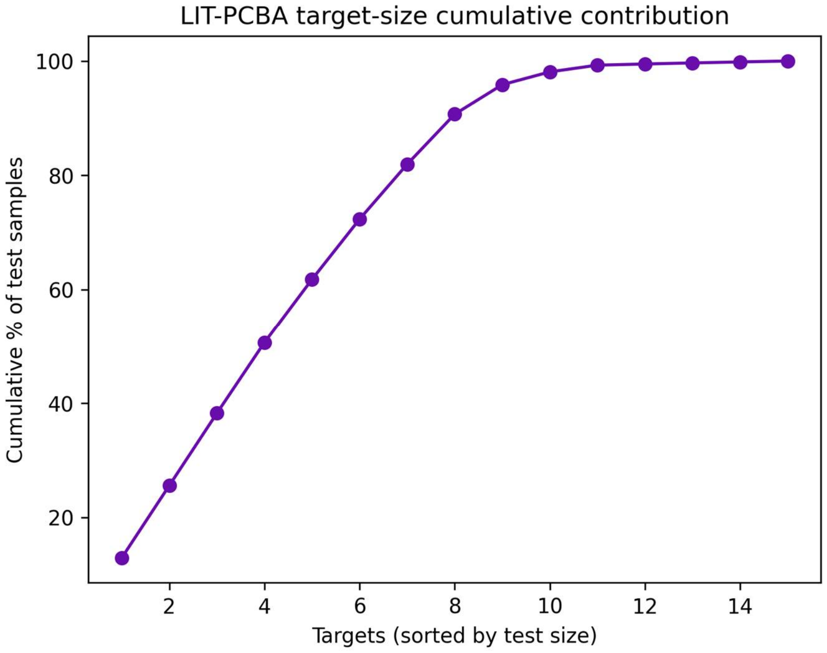

Figure S2. LIT-PCBA cumulative contribution curve

S2 Per-target Consistency

Table S3. Win/loss/tie statistics

dataset	metric	baseline	n_common	better(MA>base)	worse(MA<base)	tie
DUD-E	AUC	GNN-MA-intra	100	99	1	0
DUD-E	AUC	DeepChem	100	92	6	2
DUD-E	AUC	ECFP4	98	95	1	2
DUD-E	AUC	GMN	100	85	11	4
DUD-E	AUC	Siamese_GNN	100	95	5	0
DUD-E	EF1	GNN-MA-intra	100	95	1	4
DUD-E	EF1	DeepChem	100	15	18	67
DUD-E	EF1	ECFP4	98	94	4	0
DUD-E	EF1	GMN	100	29	14	57
DUD-E	EF1	Siamese_GNN	100	61	39	0
LIT-PCBA	AUC	GNN-MA-intra	15	15	0	0
LIT-PCBA	AUC	DeepChem	15	15	0	0
LIT-PCBA	AUC	ECFP4	15	15	0	0
LIT-PCBA	AUC	GMN	15	15	0	0
LIT-PCBA	AUC	Siamese_GNN	15	15	0	0
LIT-PCBA	EF1	GNN-MA-intra	15	14	0	1
LIT-PCBA	EF1	DeepChem	15	15	0	0
LIT-PCBA	EF1	ECFP4	15	15	0	0
LIT-PCBA	EF1	GMN	15	12	3	0
LIT-PCBA	EF1	Siamese_GNN	15	14	1	0

Table S3 summarizes cross-target consistency using win/loss/tie counts, where "better" indicates a positive per-target difference for the corresponding metric. This analysis complements macro-average and weighted-macro-average summaries by showing whether performance gains are broadly distributed across targets rather than driven by only a few cases.

S3 Global Metrics

Tables S4–S5 report global (pooled) metrics obtained by aggregating predictions across all targets. These results are provided for completeness and for comparison with pooled reporting conventions, but they should be interpreted together with macro-average and weighted-macro-average analyses because global metrics can be disproportionately affected by a small number of large targets.

Table S4. Global ROC-AUC results

dataset	model	AUC_global
DUD-E	ECFP4	0.738759
DUD-E	GMN	0.991335
DUD-E	GNN-MA	0.996843
DUD-E	GNN-MA-intra	0.917043
DUD-E	DeepChem	0.979008
DUD-E	Siamese_GNN	0.989246
LIT-PCBA	ECFP4	0.578478
LIT-PCBA	GMN	0.87976

dataset	model	AUC_global
LIT-PCBA	GNN-MA	0.924484
LIT-PCBA	GNN-MA-intra	0.507759
LIT-PCBA	DeepChem	0.912726
LIT-PCBA	Siamese_GNN	0.860938

Table S5. Global EF results

dataset	model	EF@1%_global	EF@2%_global	EF@5%_global	EF@10%_global	EF@20%_global
DUD-E	ECFP4	21.919125	13.752812	7.014234	4.355613	2.762859
DUD-E	GMN	34.220287	31.985734	18.482887	9.76106	4.970096
DUD-E	GNN-MA	35.014377	33.909595	19.654827	9.947293	4.986237
DUD-E	GNN-MA-intra	18.636284	15.552241	10.756027	7.275479	4.356749
DUD-E	DeepChem	34.815854	32.035382	17.400332	9.269407	4.826072
DUD-E	Siamese_GNN	33.976386	31.693751	18.31455	9.714972	4.946389
LIT-PCBA	ECFP4	1.834952	1.757496	1.711018	1.571463	1.437061
LIT-PCBA	GMN	13.641799	10.562156	7.63886	5.734745	4.021057
LIT-PCBA	GNN-MA	20.160294	14.986388	9.569856	7.15051	4.618056
LIT-PCBA	GNN-MA-intra	1.926428	1.624085	1.173831	1.068544	0.994624
LIT-PCBA	DeepChem	21.256811	18.550504	12.814961	7.834646	4.365157
LIT-PCBA	Siamese_GNN	10.167075	8.005375	6.969202	5.494723	3.788469

Notably, DeepChem is slightly higher than GNN-MA in pooled EF@1% on LIT-PCBA. This difference likely reflects the sensitivity of pooled evaluation to target-size imbalance, whereas GNN-MA remains clearly superior under macro-average and weighted-macro-average views.

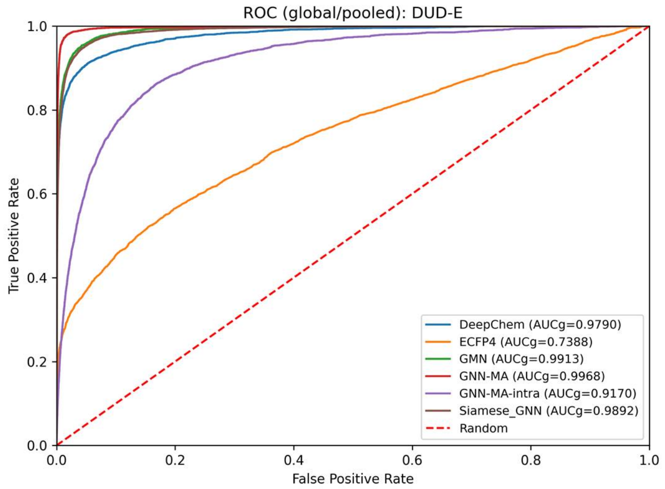

Figure S3. Global ROC curves for all compared models on DUD-E.

Figure S3 shows the pooled ROC curves of all models on DUD-E. GNN-MA achieves the best overall discrimination and outperforms GNN-MA-intra, supporting the benefit of cross-graph interaction.

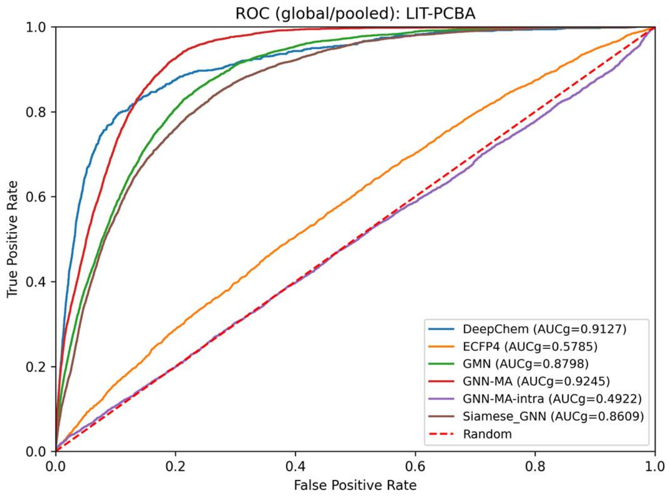

Figure S4. Global ROC curves for all compared models on LIT-PCBA.

Figure S4 shows the pooled ROC curves of all models on LIT-PCBA. GNN-MA maintains the strongest overall discrimination and remains clearly better than GNN-MA-intra, indicating the effectiveness of cross-graph soft alignment.

S4. Statistical Significance

Table S6. Statistical significance of per-target improvements

Dataset	Baseline	Metric	Mean Δ	95% CI	Wilcoxon p-value
DUD-E	MA-intra	AUC	0.1	[0.0830, 0.1177]	4.02E-18
DUD-E	MA-intra	EF@1%	18.63	[16.41, 20.90]	6.29E-18
DUD-E	MA-DeepChem	AUC	0.02	[0.0129, 0.0217]	5.90E-17
DUD-E	MA-DeepChem	EF@1%	0.57	[-0.13, 1.34]	0.8562
DUD-E	MA-ECFP4	AUC	0.24	[0.2085, 0.2616]	9.56E-18
DUD-E	MA-ECFP4	EF@1%	15.35	[12.88, 17.85]	1.66E-16
DUD-E	MA-GMN	AUC	0.01	[0.0060, 0.0122]	2.13E-14
DUD-E	MA-GMN	EF@1%	1.8	[0.73, 3.02]	0.009
DUD-E	MA-Siamese_GNN	AUC	0.01	[0.0080, 0.0178]	6.73E-17
DUD-E	MA-Siamese_GNN	EF@1%	1.88	[0.67, 3.18]	0.001
LIT-PCBA	MA-intra	AUC	0.3	[0.2751, 0.3338]	6.10E-05
LIT-PCBA	MA-intra	EF@1%	37.56	[26.04, 48.85]	7.23E-04
LIT-PCBA	MA-DeepChem	AUC	0.29	[0.2433, 0.3329]	6.10E-05
LIT-PCBA	MA-DeepChem	EF@1%	39.94	[28.76, 52.70]	6.10E-05
LIT-PCBA	MA-ECFP4	AUC	0.34	[0.3029, 0.3805]	6.10E-05
LIT-PCBA	MA-ECFP4	EF@1%	40.92	[30.72, 51.58]	6.10E-05
LIT-PCBA	MA-GMN	AUC	0.03	[0.0202, 0.0383]	6.10E-05
LIT-PCBA	MA-GMN	EF@1%	10.82	[1.78, 19.03]	0.0125
LIT-PCBA	MA-Siamese_GNN	AUC	0.06	[0.0492, 0.0736]	6.10E-05
LIT-PCBA	MA-Siamese_GNN	EF@1%	17.75	[11.13, 24.51]	1.22E-04

Table S6 reports the statistical significance of per-target improvements. Confidence intervals for the mean improvement were estimated by nonparametric bootstrap resampling over targets (B = 20,000), and p-values were obtained using a two-sided Wilcoxon signed-rank test applied to the per-target paired differences.

S5 Per-target ROC-AUC and EF@1% values

Table S7 reports the per-target ROC-AUC and EF@1% results for all compared models on DUD-E and LIT-PCBA. These values provide the raw target-level basis for the win/loss/tie summaries and statistical significance analyses reported above.

Table S7. Per-target ROC-AUC and EF@1% values for all models on DUD-E and LIT-PCBA.

		AUC						EF1					
dataset	target	GNN-MA	GNN-MA-intra	DeepChem	ECFP4	GMN	Siamese_GNN	GNN-MA	GNN-MA-intra	DeepChem	ECFP4	GMN	Siamese_GNN
	abl1	1	0.96	1	0.79	1	1	37.3	31.08	37.3	25.77	37.3	34.52
	ace	1	0.96	1	0.93	1	1	21.89	15.81	21.89	21.05	20.67	20.71
	aces	0.99	0.86	0.94	0.61	0.96	0.96	37.49	15.86	34.61	11.65	31.72	35.23
	ada	1	0.97	0.99	0.83	1	1	21.3	10.65	21.3	20.89	21.3	21.64
	ada17	1	0.98	1	0.88	1	0.99	37.77	34.71	38.79	26.41	38.79	37.47
	adrb1	1	0.86	0.98	0.83	0.99	0.98	34.98	4.12	34.98	18.94	32.92	30.64
	adrb2	1	0.9	0.99	0.74	0.99	0.99	34.17	8.54	34.17	8.68	34.17	35.5
	akt1	1	0.98	1	0.69	1	1	32.97	15.38	39.56	2.25	37.36	35.7
	akt2	1	0.96	0.99	0.63	1	1	37.63	23.52	37.63	5.17	32.93	33.53
	aldr	1	0.56	0.94	0.79	0.98	0.96	42.59	8.52	34.07	25.74	38.33	22.36
	ampc	0.97	0.61	0.92	0.86	0.9	0.74	28.38	0	14.19	41.14	28.38	36.67
	andr	1	0.93	0.99	0.7	0.97	0.96	26.6	12.42	28.38	19.15	19.51	15.24
	aofb	0.98	0.66	0.96	0.62	0.91	0.89	34.61	0	14.83	5.25	24.72	27.92
	bace1	1	0.88	0.97	0.58	0.99	0.98	38.2	2.01	34.18	1.98	28.15	29.32
	braf	1	0.99	1	0.85	1	1	39.88	36.26	39.88	22.66	39.88	32.78
	cah2	1	0.99	1	0.97	1	0.99	38.76	28.19	38.76	24.77	38.76	28.91
	casp3	0.98	0.86	0.93	0.75	0.97	0.98	31.94	0	31.94	18.67	31.94	32.83
	cdk2	0.99	0.89	0.98	0.55	0.98	0.97	33.59	14.4	34.79	0	31.19	25.99
	comt	1	0.51	1		1	1	40.4	0	40.4	0	40.4	42.89
	cp2c9	0.99	0.91	0.92	0.57	0.97	0.95	35.78	15.34	25.56	0	30.67	23.85
DUD-E	cp3a4	0.97	0.87	0.95	0.54	0.97	0.96	20.47	10.24	28.15	8.17	12.8	13.99
	csf1r	1	0.97	0.99	0.7	1	1	39.2	26.13	42.47	21.7	39.2	39.64
	cxcr4	1	0.88	1	0.57	0.99	1	27.31	6.83	27.31	13.46	27.31	23.97
	def	1	0.96	0.99	0.95	1	1	34.82	11.61	34.82	33.35	34.82	44.37
	dhi1	0.99	0.91	0.94	0.85	0.96	0.96	36.23	21.74	36.23	28.07	34.42	33.99
	dpp4	1	0.85	0.99	0.81	0.98	0.99	38.06	7.25	38.06	19.19	38.06	39.08
	drd3	1	0.86	0.98	0.57	0.97	0.98	39.43	12.05	39.43	5.6	36.14	35.35
	dyr	1	0.96	1	0.85	1	1	30.98	18.93	30.98	30.37	30.98	29.99
	egfr	1	0.96	0.99	0.89	1	0.99	42.03	29.19	43.2	35.99	43.2	39.64
	esr1	1	0.94	1	0.85	1	1	33.55	21.35	33.55	32.86	33.55	31.35
	esr2	1	0.9	0.99	0.77	1	0.99	34.87	9.96	34.87	16.36	34.87	33.38
	fa10	1	0.91	0.99	0.67	0.99	0.98	25.33	6.03	25.33	12.34	26.54	25.27
	fa7	1	0.85	1	0.89	1	1	29.32	0	34.21	34.06	29.32	31.26
	fabp4	1	0.97	1	0.97	1	0.97	41.86	27.9	41.86	41.43	41.86	44.7
	fak1	1	1	1	1	1	1	46.08	46.08	46.08	34.67	46.08	40.04
	fkb1a	1	0.96	1	0.79	1	1	21.86	15.61	21.86	14.05	21.86	20.62
	fnta	1	0.96	0.99	0.8	0.99	1	31.62	23.42	31.62	28.79	31.62	32.25
	fpps	1	1	1	1	1	1	37.72	41.91	41.91	40.27	41.91	40.18
	gcr	1	0.95	0.99		0.99	0.99	25.92	12.1	27.65	0	27.65	25.54
	glcm	1	0.97	1	0.69	1	0.99	10.42	7.82	13.03	0	13.03	10.73
	gria2	0.99	0.82	0.94	0.65	0.96	0.97	39.84	0	39.84	17.08	27.58	29.9
	grik1	1	0.66	0.96	0.8	0.97	0.99	42.44	0	42.44	36.36	12.12	18.91

hdac2	1	0.96	1	0.62	1	1	42.52	19.33	42.52	15.1	42.52	45.04
hdac8	1	0.93	1	0.87	1	1	44.83	12.23	44.83	39.55	44.83	42.82
hivint	1	0.81	0.97	0.59	0.99	0.99	31.73	0	31.73	0	27.19	34.1
hivpr	1	0.97	1	0.81	1	1	26.89	14.86	26.89	9.19	26.89	25.46
hivrt	0.99	0.74	0.88	0.56	0.96	0.94	28.92	13.7	22.83	3.09	24.36	19.06
hmdh	1	0.87	0.99	0.71	1	0.99	29.68	0	29.68	28.19	29.68	27.65
hs90a	1	0.83	0.99	0.55	0.99	0.99	32.56	0	32.56	7.17	32.56	46.18
hxk4	1	0.8	0.99	0.65	1	0.99	35.36	21.21	28.29	19.5	35.36	38.75
igf1r	1	0.99	1	0.67	1	1	36.19	32.17	40.21	4.02	40.21	39.23
inha	1	0.99	0.99	0.57	1	1	30.12	30.12	30.12	22	30.12	29.38
ital	1	0.89	0.93	0.66	0.99	1	37.21	16.54	37.21	25.27	37.21	39.17
jak2	1	0.91	0.96	0.67	0.98	0.99	42.19	36.16	42.19	12.15	36.16	34.61
kif11	1	0.89	0.96	0.84	0.96	0.97	33.95	4.24	33.95	33.76	33.95	30.28
kit	1	0.93	0.99	0.59	1	1	41.85	30.43	41.85	0	41.85	39.03
kith	1	0.79	1	1	1	1	21.57	0	21.57	20.07	21.57	24.29
kpcb	1	0.91	0.97	0.78	0.99	0.99	35.04	10.51	35.04	33.08	35.04	42.29
lck	1	0.95	0.99	0.72	1	0.99	39.96	28.55	41.39	17.17	38.54	42.53
lkha4	1	0.93	1	0.96	1	1	38.96	19.48	38.96	38.24	38.96	39.84
mapk2	1	0.89	1	0.6	1	1	29.41	16.81	29.41	0	29.41	26.94
mcr	1	0.87	0.97	0.62	0.99	0.99	27.2	18.13	27.2	8.7	27.2	23.68
met	1	0.93	0.99	0.86	1	1	46.76	27.28	46.76	45.39	46.76	40.81
mk01	1	0.94	0.99	0.76	1	1	25.55	25.55	31.93	12.43	31.93	33.2
mk10	1	0.94	0.93	0.6	0.99	0.97	34.6	24.71	34.6	4.69	34.6	29.34
mk14	1	0.94	0.99	0.78	0.99	0.99	40.61	29.92	39.54	11.84	39.54	36.23
mmp13	0.99	0.97	0.99	0.93	1	1	37.21	30.7	37.21	32.37	37.21	35.97
mp2k1	1	0.76	0.99	0.67	1	1	34	7.56	34	31.4	34	35.96
nos1	1	0.86	0.97	0.71	0.99	0.99	34.67	23.11	23.11	4.12	30.81	25.24
nram	1	0.93	1	0.78	1	1	28.13	28.13	28.13	11.8	28.13	30.23
pa2ga	1	0.87	0.96	0.62	0.97	0.98	38.29	19.14	38.29	0	12.76	33.15
parp1	1	0.91	0.99	0.82	0.99	0.98	41.59	15.6	41.59	19.61	40.29	35.49
pde5a	1	0.95	0.99	0.78	0.99	0.99	38.29	15.04	36.92	24.21	38.29	40.74
pgh1	0.97	0.77	0.87	0.51	0.91	0.95	35.93	3.59	28.74	15.24	7.19	28.29
pgh2	0.99	0.88	0.98	0.75	0.97	0.96	40.66	25.87	42.5	30.75	44.35	42.04
plk1	1	0.94	1	0.62	1	0.99	38.55	27.54	38.55	5.68	38.55	38.95
pnph	1	0.99	1	0.94	1	1	30.29	26.51	30.29	29.29	30.29	27.4
ppara	1	0.93	1	0.88	1	1	37.07	5.3	37.07	29.63	37.07	38.21
ppard	1	0.92	0.98	0.95	0.99	0.99	45.13	0	45.13	35.03	45.13	40.96
pparg	0.99	0.9	0.95	0.78	0.98	0.98	35.1	10.8	36.45	35.07	36.45	36.51
prgr	1	0.84	0.95	0.6	0.98	0.98	34.03	12.76	29.78	6.96	31.9	27.87
ptn1	1	0.89	0.99	0.63	1	0.99	33.22	12.46	33.22	15.65	29.07	32.74
pur2	1	0.94	1	1	1	1	14	0	14	13.19	14	15.08
pygm	0.99	0.88	0.97	0.81	0.92	0.95	34.75	0	27.8	0	13.9	0
pyrd	0.99	0.82	0.97	0.77	0.98	0.98	41.63	6.94	34.69	40.9	48.57	37
reni	1	0.96	1	0.7	1	1	18.48	4.62	18.48	6.67	18.48	16.97
rock1	1	0.94	0.98	0.51	0.99	0.99	31.43	22.45	31.43	8.91	31.43	27.86
rxra	1	0.87	0.99	0.85	1	1	46.41	0	46.41	0	46.41	48.7
sahh	1	1	1	1	1	1	19.37	14.53	19.37	18.32	19.37	19.97
src	1	0.97	0.99	0.57	1	0.99	41.45	33.16	42.63	15.22	41.45	42.27

	tgfr1	1	0.99	1	0.95	1	1	30.97	27.52	30.97	25.23	30.97	35.79
	thb	1	0.89	0.93	0.76	0.98	0.99	43.5	10.88	43.5	30.13	38.06	36.6
	thrb	1	0.88	0.99	0.82	1	0.99	32.41	12.29	32.41	4.54	32.41	33.97
	try1	1	0.93	1	0.82	1	1	35.06	11.69	35.06	22.95	35.06	34.55
	tryb1	1	0.83	0.98	0.89	0.99	0.99	43.89	16.46	43.89	5.2	38.4	47.48
	tysy	1	0.95	0.99	0.77	1	0.99	19.71	11.27	22.53	20.47	22.53	23.55
	urok	1	0.94	1	0.91	1	1	32.06	20.4	32.06	17.06	32.06	36.48
	vgfr2	0.99	0.96	1	0.75	1	1	40.18	22.5	40.18	19.36	41.79	40.58
	wee1	1	0.99	0.99	0.93	1	1	42.6	30.43	42.6	40.87	42.6	39.45
	xiap	1	0.78	1	1	1	0.99	38.29	0	38.29	36.64	38.29	46.22
	adrb2	1	0.62	0.72	0.53	0.96	0.89	99.97	33.32	0	20.83	62.48	69.21
	aldh1	0.99	0.74	0.61	0.67	0.97	0.96	69.3	0	0	0	42.9	28.43
	esr1_ago	0.79	0.57	0.65	0.5	0.77	0.76	9.99	1.18	4.6	0.94	7.31	5.75
	esr1_ant	0.98	0.59	0.62	0.55	0.95	0.91	20.77	0.65	0	1.42	15.58	19.6
	fen1	0.95	0.63	0.82	0.68	0.94	0.92	36.82	10.52	7.88	5.55	39.45	34.83
	gba	0.99	0.71	0.67	0.64	0.92	0.92	63.89	0	5.55	0	33.33	32.89
	idh1	0.97	0.73	0.59	0.58	0.95	0.9	59.99	6	19.99	0	37.99	30.9
LIT-PCBA	kat2a	0.85	0.53	0.61	0.57	0.79	0.76	29.98	2.5	5	5	9.99	10.64
	mapk1	0.86	0.6	0.57	0.52	0.83	0.77	12.78	8.23	3.12	0.36	9.94	7.93
	mtorc1	0.97	0.61	0.65	0.5	0.92	0.92	57.26	0	8.85	0	53.62	39.94
	oprk1	0.99	0.75	0.56	0.81	0.96	0.9	33.32	33.32	0	0	66.63	33.32
	pkm2	0.95	0.56	0.72	0.56	0.91	0.9	38.83	1.56	1.79	5.77	22.32	21.87
	pparg	0.99	0.68	0.73	0.72	0.98	0.93	63.7	2.05	0	7.39	36.98	24.45
	tp53	0.95	0.69	0.56	0.61	0.94	0.88	32.35	4.31	0	3.12	25.88	18.07
	vdr	0.92	0.58	0.74	0.59	0.9	0.89	39.3	1.12	12.34	4	41.55	24.11
